# Molecular Dynamics Simulations of the SPRED2^Leu100Pro^ EVH-1 Domain Complexed with the GAP-Related Domain of Neurofibromin

**DOI:** 10.3390/ijms26094342

**Published:** 2025-05-02

**Authors:** Martina Terrusa, Elisa Sangiovanni, Marialetizia Motta, Marco Tartaglia, Ingrid Guarnetti Prandi, Giovanni Chillemi

**Affiliations:** 1 Department for Innovation in Biological, Agro-Food and Forest Systems—DIBAF, University of Tuscia, Via S. Camillo de Lellis s.n.c, 01100 Viterbo, Italy; martina.terrusa@unitus.it (M.T.); e.sangiov@unitus.it (E.S.); ingrid.prandi@unitus.it (I.G.P.); 2 Molecular Genetics and Functional Genomics, Bambino Gesù Children’s Hospital IRCCS, 00146 Rome, Italy; marialetizia.motta@opbg.net (M.M.); marco.tartaglia@opbg.net (M.T.); 3 Department of Experimental Medicine, University of Rome “Tor Vergata”, 00133 Rome, Italy; 4 National Institute for Infectious Diseases “Lazzaro Spallanzani”—IRCCS, 00149 Rome, Italy

**Keywords:** sprouty-related EVH-1 domain-containing, neurofibromatosis type I, RAS signaling, Noonan syndrome, molecular dynamics, essential dynamics

## Abstract

The homozygous Leu100Pro amino acid substitution in SPRED2, a protein negatively controlling RAS function, has recently been identified to be causally linked to a recessive form of Noonan syndrome. The amino acid substitution was documented to affect protein stability and cause a decreased and/or less stable interaction with neurofibromin, a RAS-specific GTPase activating protein negatively regulating RAS function. To further investigate the structural and functional impact of Leu100Pro, we structurally characterized the consequences of this change on the interaction of SPRED2 with neurofibromin, by 1 µn-long molecular dynamics (MD) simulations. Our analyses failed in identifying local perturbations predicted to disrupt or dramatically affect SPRED2 binding to neurofibromin, though a rearrangement of their interaction was observed. On the other hand, MD simulations also identified long-range structural rearrangements of the SPRED2 EVH-1 domain, which might be relevant for an aberrant folding of the mutant driving the previously documented accelerated degradation. Overall, the performed MD simulations suggest the occurrence of multiple intramolecular and intermolecular structural perturbations driven by the Leu100Pro change that likely contribute to its LoF behavior.

## 1. Introduction

**Figure 1:**
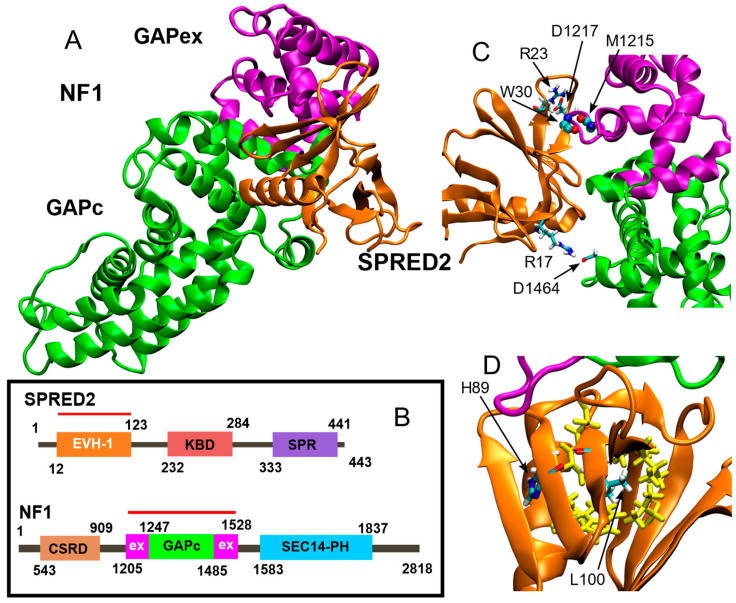
The neurofibromin-SPRED2 complex as sampled by the MD simulation. (**A**) Centroid of the most populated cluster, showing GAPc in green, GAPex in purple and SPRED2 EVH-1domain in orange. (**B**) Domain organization with modeled regions highlighted by a red line. (**C**) Representative intermolecular H-bonds of the WT system. (**D**) Leu100 located in a β-Sheet (central to a yellow highlighted hydrophobic cluster) forms a stabilizing H-bond with His89 (see Table 1).

Sprouty-related EVH-1 domain-containing (SPRED) proteins constitute a family of regulators that negatively control the RAS-mitogen-activated protein kinase (MAPK) signaling pathway [1]. The three paralogous, *SPRED1* (MIM: 609291), *SPRED2* (MIM: 609292) and *SPRED3* (MIM: 609293), share a similar domain organization and partial functional overlap [2]. These proteins consist of an *N*-terminal Ena/Vasp homology (EVH-1) domain, a c-KIT binding domain (KBD) (not occurring in SPRED3), and a *C*-terminal sprouty related (SPR) domain (Figure 1B) [2]. Their negative role in RAS-MAPK signaling modulation is attained by their ability to bind to neurofibromin, a RAS-specific GTPase activating protein (GAP), favoring its recruitment to the plasma membrane [2].

Heterozygous loss-of-function (LoF) variants in *SPRED1* underlie Legius syndrome (LS, MIM: 611431) [3], a disorder having some similarities to neurofibromatosis type I (NF1, MIM: 162200) [4], which is caused by inactivating heterozygous mutations in *NF1* (MIM: 613113) [5], the gene encoding neurofibromin [6]. Notably, biallelic LoF variants in *SPRED2* underlie a recessive form of Noonan syndrome (NS; MIM: PS163950) [7,8]. NS, LS, and an increasing number of clinically related disorders are collectively known as RASopathies as they share upregulated signaling through RAS as a common mechanism of disease [9]. In these conditions, enhanced signaling through the RAS-MAPK cascade can result from the functional upregulation of various RAS GTPases (i.e., HRAS, KRAS, NRAS, RIT1, MRAS, RRAS and RRAS2), enhanced function of various proteins positively controlling RAS function or favoring the RAS-mediated activation of downstream transducers (i.e., SHP2, SOS1, SOS2, SHOC2 and PPP1CB), functional upregulation of RAS effectors participating in the MAPK cascade (i.e., BRAF, RAF1, MAP2K1, MAP2K2 and MAPK1), or inefficient RAS-MAPK signaling switch-off due to the impaired function of negative regulators (i.e., CBL, neurofibromin, SYNGAP, SPRED1, SPRED2 and LZTR1) [9].

While the regulatory role of SPRED1 and SPRED2 in controlling RAS function and MAPK signaling is now well recognized [2,10,11], and the functional mechanisms by which LS-causing *SPRED1* variants cause LoF of the protein have been characterized [12,13], the precise events by which the disease-causing *SPRED2* variants perturb protein function have not been fully investigated. Indeed, the overall effect of these variants has been shown to converge toward the upregulation of MAPK signaling, and impact on SPRED2 function, by affecting protein stability, proper targeting of the protein to the plasma membrane or its binding to neurofibromin [7]. Among these recently identified pathogenic variants, the Leu100Pro substitution was documented to cause accelerated degradation of the protein and a reduced binding to the GAP [7]. Notably, inspection of the SPRED2 EVH-1 domain bound to neurofibromin, generated by homology modeling from the available SPRED1 complex [11], documented a possible impact of the Leu-to-Pro substitution at codon 100 on SPRED2 binding to neurofibromin [7].

Molecular dynamics (MD) is a computational technique able to shed light on the structural and dynamic perturbations that can be caused by the introduction of amino acid changes in a protein [14]. MD, combined with complementary techniques such as homology modeling, and quantum mechanics/molecular mechanics methods, has greatly advanced our understanding of biological processes and the functional roles of proteins [15]. Of note, large concerted protein structural rearrangements, often linked to biological functions, are always disguised in the trajectories sampled by MD by a great number of small irrelevant fluctuations. Essential dynamics (ED), related to principal component analysis, extracts such large concerted motions by diagonalizing the covariance matrix generated on the c-alpha protein atoms [16]. This approach has successfully been applied to the structural and dynamic characterization of a large number of protein systems [17].

In this work, we applied MD and ED to further investigate the structural and dynamic perturbations caused by the Leu100Pro substitution affecting the EVH-1 domain of SPRED2.

## 2. Results

Starting from the crystal structure of the ternary complex between the SPRED1 EVH-1 domain, GTPase-activating protein (GAP)-related domain (GRD) of neurofibromin and KRAS [11], we modeled the EVH-1 domain of SPRED2 interacting with the GAP-related domain of neurofibromin (Figure 1A). The domain organization of both proteins, including the GAPc (catalytic) region of neurofibromin, is shown with modeled regions highlighted by a red line (Figure 1B).

Similar to what observed for SPRED1, the EVH-1 SPRED2 domain interacts with the GAP-related domain of neurofibromin, through its N- and C-terminal regions (GAPex). In particular, our MD simulation shows a highly stable interaction between the main chain atoms of SPRED2 Trp30 and neurofibromin Met1215 (Figure 1C), which was observed for 61% of the simulation time. More variable interactions involving the side chains of SPRED2 Arg17 and Arg23 with residues Asp1217 and Asp1464 of neurofibromin were also observed, respectively (30% and 25% of the simulation time) (Figure 1C).

**Figure 2:**
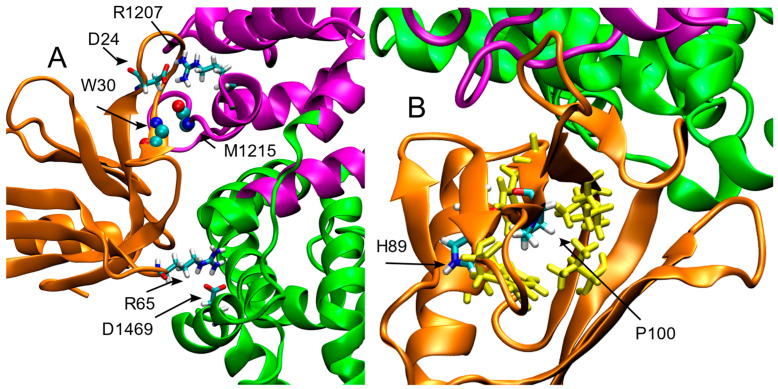
The neurofibromin-SPRED2 complex as sampled by the MD simulation of the SPRED2 L100P mutant (centroid of the most populous cluster). Colors are as in Figure 1. (**A**) Relevant interactions between SPRED2 and the GAP-related domain of neurofibromin, including a main-chain H-bond between Trp30 and Met1215, as well as side-chain H-bonds involving Asp24–Arg1207 and Arg65–Asp1469. (**B**) Pro100 abolishes the main H-bond with His89 and perturbs the conformation of the antiparallel β-sheet while the hydrophobic cluster (highlighted in yellow) remains intact.

In the MD simulation of the WT system, Leu100 forms a highly stable main chain intramolecular H-bond with His89, and is located at the center of a hydrophobic cluster composed of Ala18, Val20, Val41, Trp91, Phe102, and Phe112 (in yellow in Figure 1D). Based on the static model of Leu100 in the crystal structure, we hypothesized that the effect of the Leu-to-Pro substitution would affect the binding of SPRED2 to neurofibromin. While the occurrence of such perturbation cannot be ruled out, it was not observed during the simulated trajectory. Our findings from the MD simulations offer a different perspective of the structural perturbations introduced by the Leu-to-Pro substitution. Specifically, the H-bond between the main chain atoms of SPRED2 Trp30 and neurofibromin Met1215, in fact, is still observed, although for a reduced simulation time (40%; Figure 2A). Additionally, different side-chain interactions are observed, particularly between Arg and Asp residues, showing the extensive electrostatic complementarity between the two proteins. For instance, the interaction between SPRED2 Arg23 and Asp1217 of neurofibromin observed in the WT system, was replaced by a new interaction between Asp24 (SPRED2) and Arg1207 (neurofibromin), observed for the 65%of simulation time. Moreover, a side-chain H-bond between SPRED2 Arg65 and neurofibromin Asp1469 was also detected, with an occupancy of 20%, highlighting additional compensatory contacts emerging in the mutant complex.

**Figure 3:**
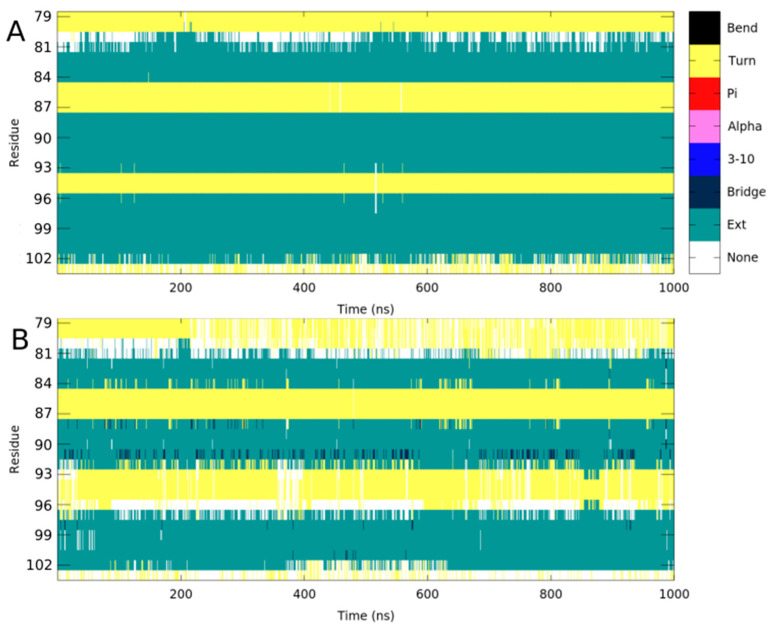
Secondary structure in the β-Sheet region of residue 100 for WT and Leu100Pro systems is shown in (**A**) and (**B**), respectively. The Ext secondary structure is stable in the WT protein, while it is strongly perturbed in the L100P mutant.

The main local perturbation introduced by the Pro100 residue is the abolition of the backbone interaction with His89, which plays a crucial role in maintaining the interaction between its main chain and this residue, contributing to proper orientation of the β-Sheet (Table 1; Figure 2B). The presence of proline, which lacks an amide group necessary for hydrogen bond formation, disrupts the local stabilization. Consequently, the antiparallel β-sheet containing Pro100, exhibits structural perturbations across all three β-strands. This effect is particularly evident when comparing the secondary structure composition of the WT and mutated systems in this region (Figure 3, panels A and B). The Ext secondary structure sheets (defined as “extended strand in parallel and/or anti-parallel β-sheet conformation”) are stably maintained in the WT but is lost in the mutant structure. This destabilization may influence the packing of the EVH-1 domain, potentially altering its interaction with neurofibromin.

Despite this structural alteration, the hydrophobic cluster surrounding residue 100, including Ala18, Val20, Val41, Trp91, Phe102, and Phe112 (in yellow in Figure 1D and Figure 2B), is conserved in the mutated system, suggesting that the overall hydrophobic core organization is preserved and potentially mitigates the destabilizing effects of the mutation on the local fold.

### 2.1. Per-Residue Root Mean Square Fluctuations

**Figure 4:**
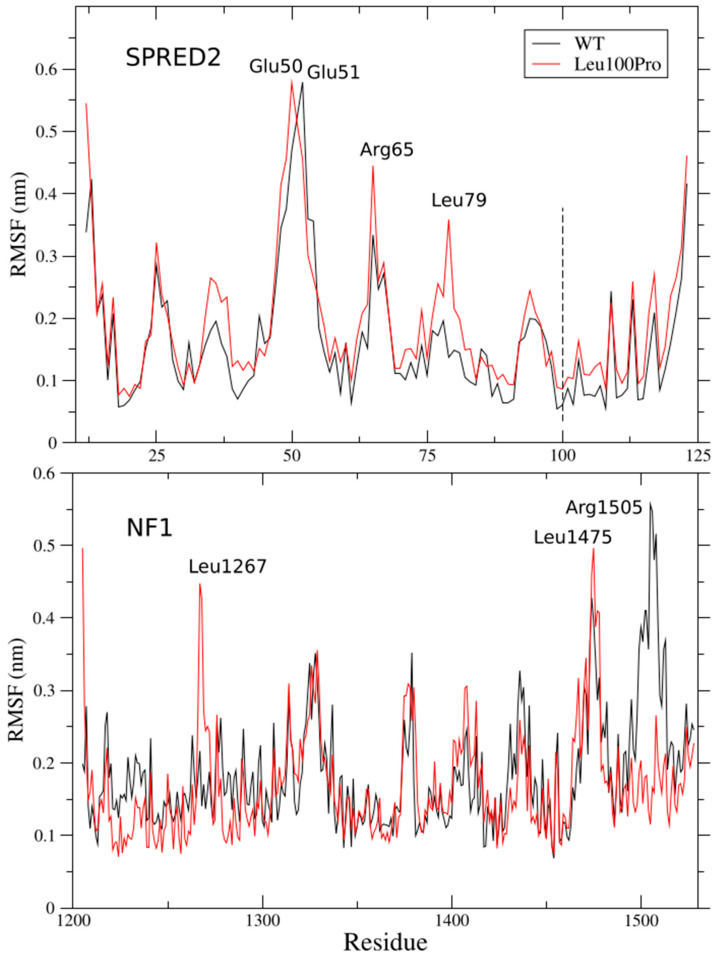
Root Mean Square Fluctuations (RMSF) of SPRED2 (**top** panel) and neurofibromin (**bottom** panel). The WT and Leu100Pro mutant are colored in black and red, respectively. The mutation site is marked with a dashed line; relevant fluctuation peaks are indicated.

In order to characterize the global effect of the Leu100Pro substitution on the SPRED2-neurofibromin complex, we analyzed the per-residue Root Mean Square Fluctuations (RMSF) of both proteins. Comparison of the RMSF profiles between the WT and mutated systems shows that the Leo100Pro substitution increases the fluctuations of SPRED2 in several regions, even those distant from the mutation site. While the highest fluctuation peak is maintained around residues 50–52, an increase in mobility is observed, for example, in the Arg65 and Leu79 regions (Figure 4, upper panel). Notably, Arg65 has been observed to form a side chain interaction with Asp1469 of neurofibromin (Figure 2A), suggesting that the amino acid substitution may indirectly influence SPRED2-neurofibromin interactions.

The effects of the Leu100Pro substitution extend beyond SPRED2. An increase in fluctuation was observed in the regions of neurofibromin flanking Leu1267 and Leu1475, while the RMSF peak in the Arg1505 region of the WT system was completely abolished by mutation (Figure 4, lower panel). These results indicate that the Leu100Pro change not only disrupts local interactions but also induces long-range structural perturbations.

### 2.2. Large Collective Protein Movements

RMSF analysis alone are not capable of revealing “large collective protein movements” that often linked to biological functions. In this regard, essential dynamics (ED) analysis, is able to separate these large collective protein movements from the “small uninteresting motions”, which are uncorrelated with other protein motions and mask the important functional one [16]. ED is usually applied only to the c-alpha atoms, as they effectively describe the motion of the protein backbone [16]. We performed ED separately on the c-alpha atoms of SPRED2 and neurofibromin to highlight how the Leu100Pro change altered the intra-protein collective movements.

**Figure 5:**
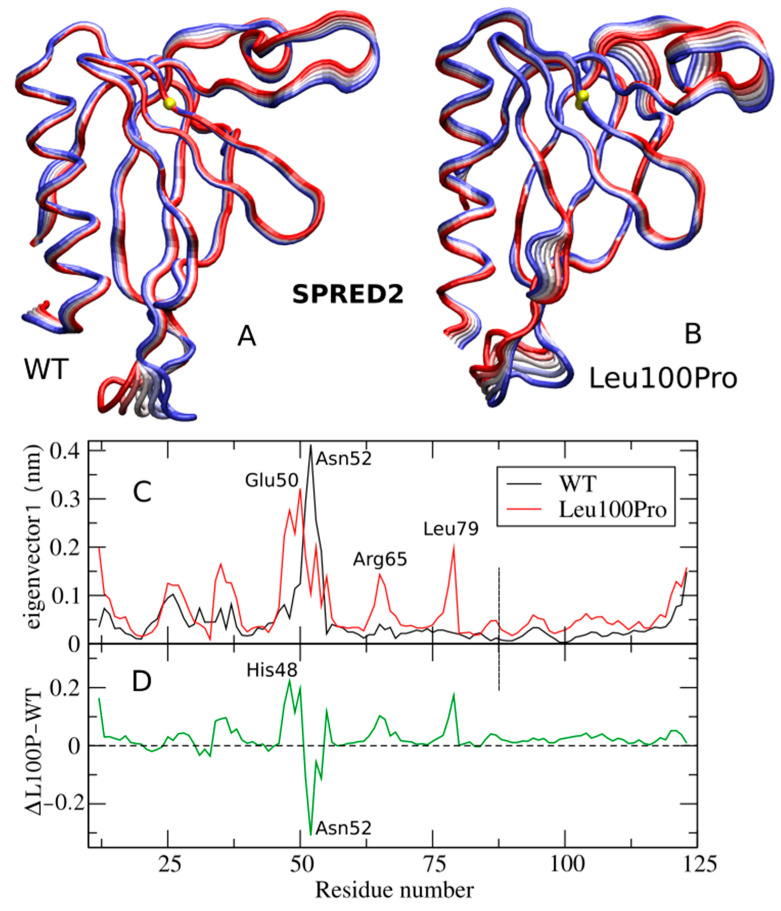
Large collective protein movements of the SPRED2 protein (WT and Leu100Pro systems) along ED eigenvector 1. (**A**,**B**) 3D projections of the WT and Leu100Pro trajectories; extreme projections are colored in blue and red, with the average structure in white. Residue 100 is represented as a yellow sphere. (**C**) RMSF of the filtered trajectories for the WT and Leu100Pro SPRED2 systems with peaks labeled by residue number. (**D**) Difference of filtered RMSF between L100P and WT systems.

ED analysis of SPRED2 is shown in Figure 5, depicting the filtered trajectories along eigenvectors 1 for both the SPRED2 WT and Leu100Pro systems. This analysis reveals that the three regions exhibiting increased RMSF in the mutant (i.e., Glu50, Arg65 and Leu79 ones in Figure 5A, red line) also show increased fluctuations along the ED filtered trajectory (Figure 5C). This indicates that the large-scale collective protein motion is strongly altered by the Leu100Pro substitution. This rearrangement can also be appreciated from the 3D structures in Figure 5A,B that compare the motion along eigenvector 1 in the WT and Leu100Pro systems, respectively (see also Supplementary Movie).

The most perturbed region is located around residues 48–52, as highlighted by the plot of the difference between the curves of Figure 5C, shown in Figure 5D. His48, in fact, exhibits the largest increase in RMSF along eigenvector 1, whereas Asn52 shows the greatest reduction.

**Figure 6:**
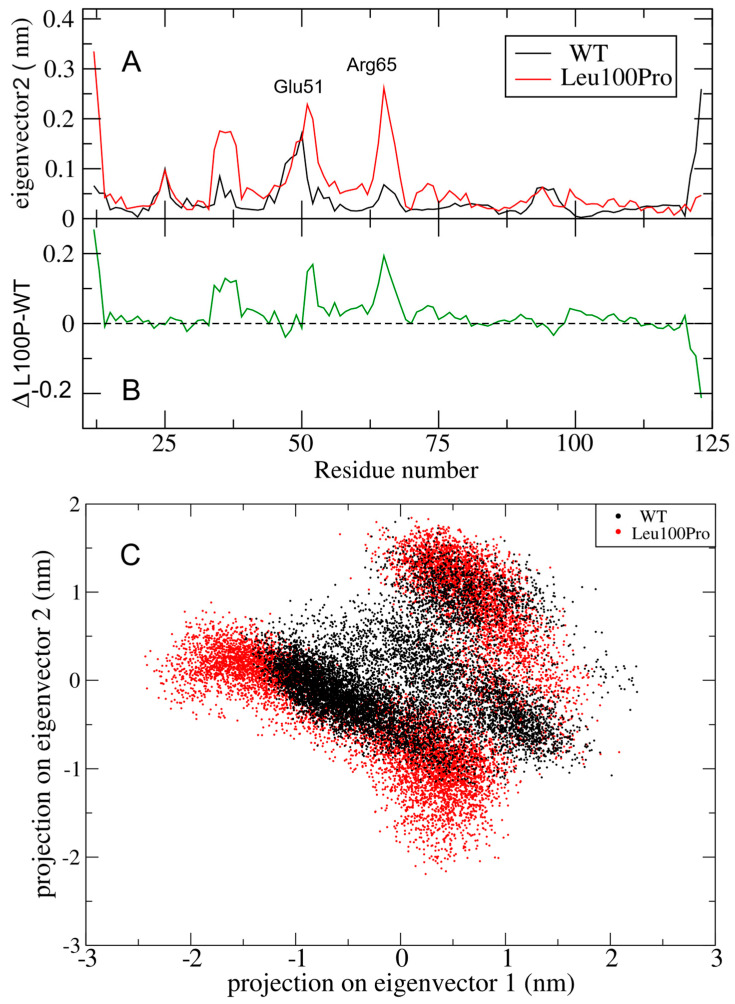
Large collective protein movements of SPRED2 protein WT and Leu100Pro systems along ED eigenvector 2. (**A**) RMSF in nm of WT and Leu100Pro filtered trajectories (black and red respectively), with fluctuation peaks labeled. (**B**) Difference of filtered RMSF of the two systems. L100P refers to the Leu100Pro substitution in SPRED2; Δ indicates the difference between Leu100Pro and WT values. (**C**) 2D projection of WT (black) and Leu100Pro systems (red) trajectories in the essential subspace of eigenvectors 1 and 2.

The regions around Glu50 and Arg65 show an increment in RMSF also along eigenvector 2 (Figure 6A), albeit no peaks show a difference greater than 0.2 nm (Figure 6B). The altered essential motion in the mutant system is further evident in the 2D projections of the trajectories within the essential subspace of eigenvectors 1 and 2 (Figure 6C). The essential subspace sampled by the Leu100Pro mutant (red in Figure 6C) is not only distinct but also broader than that explored by WT, indicating enhanced conformational variability.

## 3. Discussion

We performed MD simulations of the SPRED2 EVH-1 domain-neurofibromin GAP-related domain complex to further investigate the structural consequences of Leu100Pro, a substitution that was recently causally associated with a recessive form of NS, on the SPRED2-neurofibromin interaction [7]. Based on the original 3D model, we originally hypothesized that this change would have the ability to perturb both the interaction with neurofibromin and the integrity of the hydrophobic cluster surrounding Leu100. Unexpectedly, in the performed 1 µn-MD simulation the SPRED2-neurofibromin complex was observed to retain major interactions, albeit through a structural rearrangement of the interacting surfaces. Differently, the Leu-to-Pro substitution was observed to disrupt the backbone interaction with His89, leading to long-range intramolecular perturbations. These effects were captured by ED analysis, which highlighted significant alterations in the large collective motions along eigenvectors 1 and 2.

We previously showed that the Leu100Pro substitution causes accelerated protein degradation and affected binding of SPRED2 to neurofibromin, impairing EGF-dependent translocation of the latter to the plasma membrane. The amino acid substitution was not observed to impair proper SPRED2 targeting to the plasma membrane. The present MD simulations highlight long-range structural effect promoted by the Leu100Pro substitution, which might be relevant for an aberrant folding of the mutant driving accelerated degradation. While, the present analyses did not provide evidence of a substantial structural reorganization of the EVH-1 domain of the mutant impacting neurofibromin binding, our findings document a structural rearrangement of the SPRED2/neurofibromin interacting surfaces. We cannot exclude, therefore, that longer simulation times may lead to the loss of interaction between the two proteins.

Overall, in line with the available experimental evidence, the performed MD simulations suggest the occurrence of multiple intramolecular and intermolecular structural perturbations driven by the Leu100Pro change that likely contribute to its LoF behavior.

## 4. Materials and Methods

### 4.1. Molecular Model

The system under analysis, SPRED2 bonded with neurofibromin, was extracted from the 6v65 PDB X-ray diffraction structure of the KRAS-neurofibromin-SPRED1 complex at 2.76 Å resolution [11]. In the complex, both the neurofibromin GAP-related domain (GRD) and the enabled/vasodilator-stimulated phosphoprotein homology-1 (EVH-1) domain of SPRED1 are resolved. EVH-1 is a highly conserved domain throughout the SPRED family [10] and in the residue range of the model (13–124 in SPRED1), SPRED1 and 2 are identical with residue numbering of SPRED2 shifted by minus 1 (residues 12–123).

The PDB structure required some refinement due to missing residues from the crystal structure. These refinements were resolved by using the SWISS-MODEL automated protein structure homology modeling server [18]. The protein structure was then visualized using the VMD visualization software (Version 1.17.3) [19] and the UCSF Chimera software (Version 1.17.3) [20].

### 4.2. Molecular Dynamics Simulation of the Wild-Type System for Equilibration

The system for MD simulation was prepared using GROMACS 2020.3 software [21] to stabilize the crystal-refined structure. The WT SPRED2-neurofibromin complex was placed in a cubic box, solvated with water (TIP3P water model) [22] and neutralized by adding 2 Cl^−^ using the GROMACS tools: editconf, solvate and genion. The Charmm36 force field was employed to describe the system [23]. The protein simulation box was minimized with both the steep descendent algorithm and then the conjugate gradient algorithm. After that, 500 ns simulation was performed with a 2 fs time step, using GROMACS 2020.3. The Linear Constraint Solver (Lincs) algorithm [24] was applied to constrain bond involving hydrogen atoms. The simulation was executed on the high-performance computing m100 system at CINECA center, Bologna, Italy, using the bioinformatics Elixir-IT resources on g100 at CINECA for the analysis [25]. Long-range interactions in the system were described using the Particle mesh Ewald (PME) method [26]. During the simulation, the velocity rescaling thermostat was applied to maintain a constat temperature of 300 K [27].

### 4.3. Mutation Insertion and Trajectory Production

At the end of the 500 ns WT MD simulation, the structure was mutated using the ‘rotamers’ tool in UCSF Chimera [20] to replace the Leu residue at position 100 by Pro. After choosing the side chain position with highest probability, local clashes in the Pro side chain were resolved using the ‘minimize structure’ function of UCSF Chimera [20]. This tool performs a local minimization of 10 steps using the steepest descendent algorithm to optimize the Pro surroundings. The Leu100Pro system were equilibrated with the same GROMACS protocol discussed above. A production simulation of both the WT and L100P systems was then conducted for 1000 ns, using the same GROMACS protocol previously described, and the resulting trajectories were analyzed accordingly.

### 4.4. Molecular Dynamics Analyses

The GROMACS cluster tool [21] was applied to both WT and Leu100Pro using an RMSD cutoff of 0.25 nm on the 10,000 frames extracted along the entire trajectory. The most populated cluster of the WT and Leu100Pro systems contains 75.4% and 80% of the analyzed structures, respectively. The centroids of these clusters, corresponding to simulation time of 420.9 and 472.5 ns, respectively, are visualized in Figure 1 and Figure 2.

The per-residue Root Mean Square Fluctuations (RMSF) have been calculated using the GROMACS rmsf tool and plotted with the Xmgrace software (Version 5.1.25). The H-bond analyses for the residue 100 and the SPRED2-NF1 interaction surface were performed using the VMD ‘hydrogen-bonds’ tool [19]. The secondary structure was calculated with the VMD ‘timeline’ tool and then, thanks to bash ad-hoc script, visualized through gnuplot software (Version 6.0). ED analysis was performed with GROMACS covar and anaeig tools on the c-alpha atoms of the SPRED2 system [16].

## Figures and Tables

**Table 1:** Hydrogen bond interaction of residue 100 in the WT and mutated systems.

	Donor	Acceptor	Occupancy
WT	His89-Main-N	Leu100-Main-O	44.0%
	Leu100-Main-N	His89-Main-O	43.4%
L100P	His89-Main-N	Pro100-Main-O	6.2%

## Data Availability

Data is contained within the article or Supplementary Material.

## Supplementary Materials

The following supporting information can be downloaded at: https://www.mdpi.com/article/10.3390/ijms26094342/s1.

## Abbreviations

The following abbreviations are used in this manuscript:

SPRED	Sprouty-related EVH-1 domain-containing proteins
EVH-1	Enabled/Vasodilator-stimulated phosphoprotein homology-1
KBD	c-KIT Binding Domain
SPR	Sprouty-Related
LoF	Loss-of-Function
LS	Legius Syndrome
NS	Noonan Syndrome
MD	Molecular Dynamics
MAPK	Mitogen-Activated Protein Kinase
GAP	GTPase-Activating Protein
GAPex	GAP External region
RMSF	Root Mean Square Fluctuation
ED	Essential Dynamics
WT	Wild-type
Lincs	Linear Constraint Solver
PME	Particle Mesh Ewald
