# Supplementary figures and images for: Molecular Dynamics Simulations of the SPRED2Leu100Pro EVH-1 Domain Complexed with the GAP-Related Domain of Neurofibromin
Source: Int J Mol Sci. 2025 May 2;26(9):4342. doi: 10.3390/ijms26094342 (PMC12072562; doi:10.3390/ijms26094342)

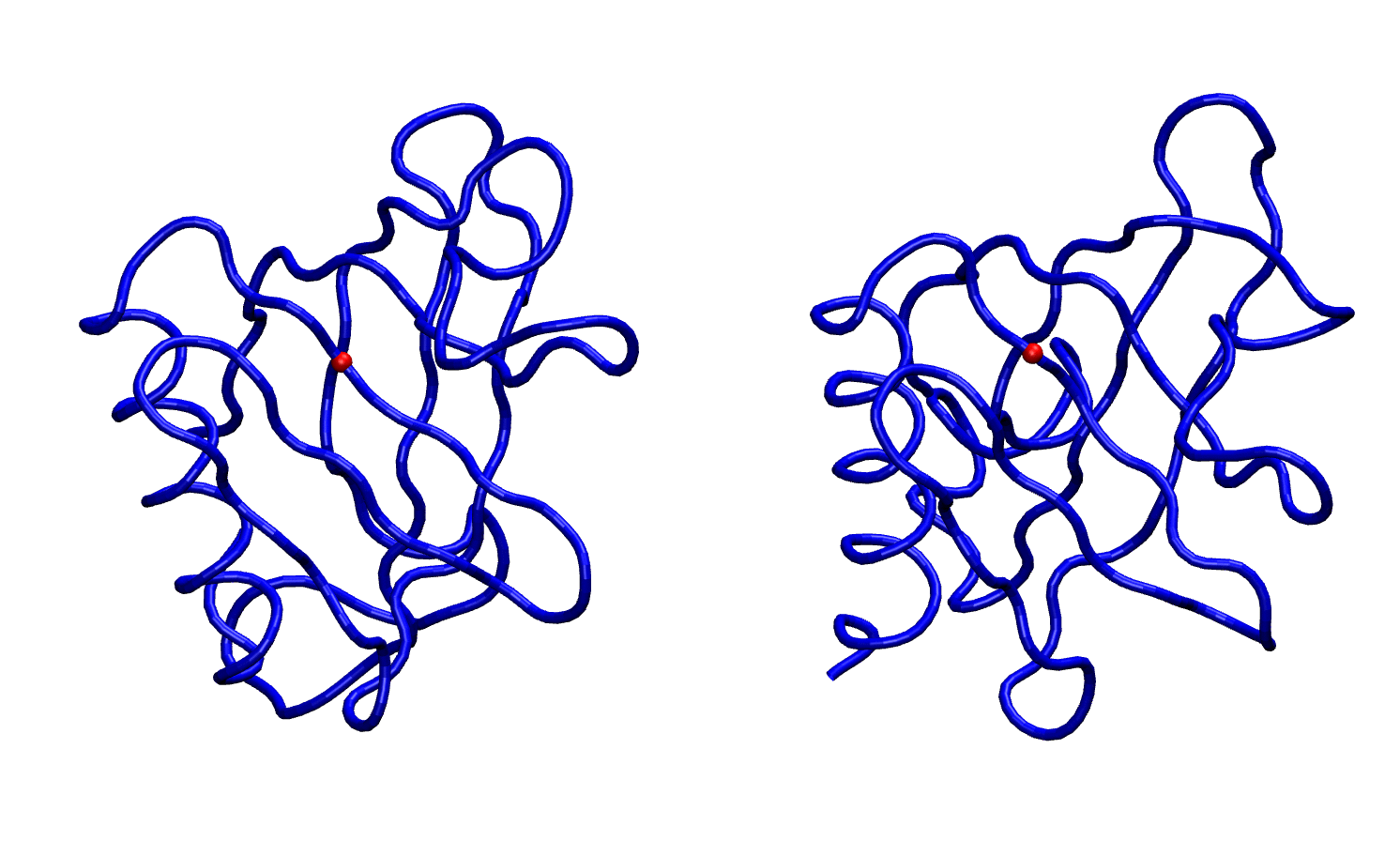

Supplement: Supplementary file 1 [file ijms-26-04342-s001.zip › ijms-3582671-supplementary.gif]
